# Characterization of the complete mitochondrial genome and phylogenetic analysis of *Menochilus sexmaculata* (Fabricius, 1781) (Coleoptera: Coccinellidae)

**DOI:** 10.1080/23802359.2021.1964401

**Published:** 2021-08-18

**Authors:** Zhifu Cui, Fengqin Cao, Zhengqiang Peng

**Affiliations:** a College of Tropical Crops, Hainan University, Haikou, China; b College of Plant Protection, Hainan University, Haikou, China; c Environment and Plant Protection Institute, Chinese Academy of Tropical Agriculture Sciences, Haikou, China

**Keywords:** *Menochilus sexmaculata*, Coccinellidae, mitochondrial genome, phylogenetic analysis

## Abstract

We sequenced the complete mitochondrial genome of *Menochilus sexmaculata* (Fabricius, 1781) and compared it with that of other insects. The mitogenome of *M. sexmaculata* is a circular molecule of 16,663 bp with 75.00% AT content, containing 13 protein-coding genes (PCGs), 22 tRNAs, 2 rRNAs, and one non-coding control region. All of the PCGs use the typical ATN as the initiation codon, with the exception of cox1 and nad3 which begin with AAT and TTG, respectively. Cox1, cox2, cox3, nad3, nad4, nad5 and nad6 employ a single T as a termination signal, while others have the typical termination codons (TAA or TAG). All the 22 typical animal tRNA genes are found in *M. sexmaculata* mitogenome, and most of the tRNAs could be folded into the classic cloverleaf secondary structure. Phylogenetic tree based on 13 PCGs suggested that *M. sexmaculata* is closely related to *Anatis ocellata* and *Calvia championorum*, and clustered within Coccinellidae.

The coccinellid beetles, *Menochilus sexmaculata* (Coleoptera: Coccinellidae), is one of the most important predator of mustard aphids, which is of great significance for the prevention and control of aphids on farm crops, fruits and vegetables (Indumathi and Savithri 2013). It can prey on more than a dozen species of aphids such as *Aphis gossypii*, *Aphis craccivora*, and *Myzus persicae* (Choudhury and Pal 2006). In this study, we sequenced and annotated the complete mitogenome of *M. sexmaculata.*

Adult individuals of *M. sexmaculata* were collected from Vegetable Base of Hainan University, Hainan, China (20°03′23″N, 110°19′44″E). Samples were preserved in 95% ethanol and stored in the College of Tropical Crops Hainan University with an accession number HU-2021-00010 (URL, Cui zhifu, zhifu555@163.com). Total genomic DNA of *M. sexmaculata* was extracted by CTAB method (Reineke et al. 1998). The mitogenome sequence was generated using Illumina HiSeq X TEN Sequencing System with 150 bp paired-end reads. The complete nucleic acid sequence was assembled by the MITObim software (Hahn et al. 2013) with the complete mitogenome (NC_046481) of *Hippodamia variegata* as a reference. The annotations were mainly compared with the existing mitochondrial genomes of related species, and the annotation results were confirmed by MITOS webserver (Bernt et al. 2013).

We obtained the complete mitogenome of *M. sexmaculata*, with 16,663 bp long (GenBank accession number MZ334467). This mitogenome encoded 13 protein-coding genes (PCGs), 22 transfer RNA genes (tRNAs), two ribosomal RNA unit genes (*rrnL* and *rrnS*), and one non-coding control region. The order and orientation of the mitochondrial genes were identical to the inferred ancestral arrangement of insects (Boore 1999). Gene overlaps were found at 10 gene junctions and involved a total of 36 bp, with two longest overlaps (8 bp) between *trnW* and *trnC*, whereas a total of 719 bp intergenic spacers were presented in eight positions, ranging in size from 1 to 684 bp. 23 genes were transcribed on the majority strand (J-strand), while the others were encoded on the minority strand (N-strand).

The *M. sexmaculata* mitogenome with an A + T content of 75.00% showed a negative AT-skew (-0.094) and a positive GC-skew (0.018). Among the 13 PCGs, the lowest A + T content was 70.53% in *cox1*, while the highest was 85.24% in *nad6*. Eleven PCGs started with a typical ATN codon: four (*nad2*, *atp8*, *nad1*, and *nad5*) with ATT, two (*cox2* and *nad6*) with ATA, five (*atp6*, *cox3*, *nad4*, *nad4L* and *cob*) with ATG. *Cox1* with ATT, and *nad3* with TTG. Six PCGs terminate with conventional stop codons (TAA and TAG), while seven genes (*cox1*, *cox2*, *cox3*, *nad3*, *nad4*, *nad5* and *nad6*) use incomplete codon (T) as termination codon.

The total length of 22 tRNAs is 1,405 bp in the mitogenome of *M. sexmaculata*, with sizes ranging from 60 bp (*trnP*) to 70 bp (*trnK*), A + T content ranged from 53.97% (*trnI*) to 92.06% (*trnE*). All the tRNA genes can be folded into the typical clover-leaf secondary structure, except for *trnS1*, which lack dihydrouridine (DHU) arm. It is a common phenomenon that *trnS1* lacks DHU arm in the mitochondrial genome of many insects (Xiong et al. 2019).

Based on the concatenated amino acid sequences of 13 PCGs from 13 Coccinellidae species, the maximum likelihood method was used to construct the phylogenetic relationship. *Drosophila melanogaster* was used as an outgroup. The result showed that *M. sexmaculata* is closely related to *Anatis ocellata* and *Calvia championorum*, and clustered within Coccinellidae, which accord with the traditional taxonomy (Figure 1). In conclusion, the complete mitogenome of *M. sexmaculata* is decoded in this study and provides essential and important DNA molecular data for further phylogenetic and evolutionary analysis for Coccinellidae.

**Figure 1.**
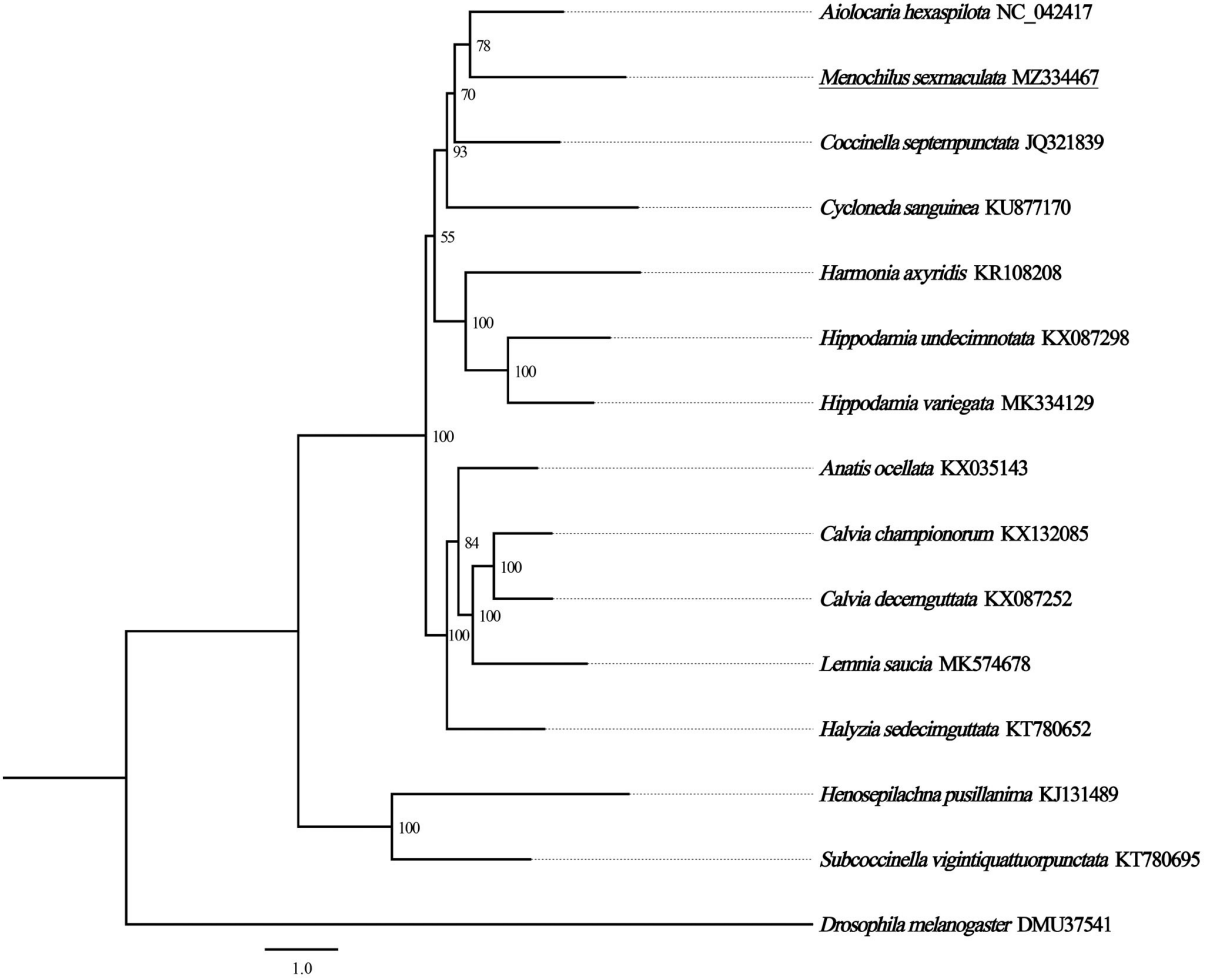
Phylogenetic tree showing the relationship between *Menochilus sexmaculata* and 14 other species based on maximum likelihood method. GenBank accession numbers of each species were listed in the tree. The coccinellid beetles determined in this study has been underlined. Numbers on branches are bootstrap values. *Drosophila melanogaster* was used as an outgroup.

## Data Availability

The genome sequence data that support the findings of this study are openly available in GenBank of NCBI at (https://www.ncbi.nlm.nih.gov/) under the accession no. MZ334467. The associated BioProject, SRA, and Bio-Sample numbers are PRJNA737735, SRR14817169, and SAMN19707761, respectively.
